# Relative Contributions of Geographic, Socioeconomic, and Lifestyle Factors to Quality of Life, Frailty, and Mortality in Elderly

**DOI:** 10.1371/journal.pone.0008775

**Published:** 2010-01-19

**Authors:** Jean Woo, Ruth Chan, Jason Leung, Moses Wong

**Affiliations:** 1 Department of Medicine and Therapeutics, Chinese University of Hong Kong, Hong Kong, China; 2 Centre for Nutritional Studies, School of Public Health, Chinese University of Hong Kong, Hong Kong, China; 3 Jockey Club Centre for Osteoporosis Care and Control, Chinese University of Hong Kong, Hong Kong, China; The Kenya Medical Research Institute, Kenya

## Abstract

**Background:**

To date, few studies address disparities in older populations specifically using frailty as one of the health outcomes and examining the relative contributions of individual and environmental factors to health outcomes.

**Methodology/Principal Findings:**

Using a data set from a health survey of 4,000 people aged 65 years and over living in all regions of Hong Kong, we examined regional variations in self-rated health, frailty, and four-year mortality, and analyzed the relative contributions of lifestyle, socioeconomic status, and geographical location of residence to these outcomes using path analysis. We hypothesize that lifestyle, socioeconomic status, and regional characteristics directly and indirectly through interactions contribute to self-rated physical and psychological health, frailty, and four-year mortality.

District variations directly affect self-rated physical health, and also exert an effect through socioeconomic position as well as lifestyle factors. Socioeconomic position in turn directly affects self-rated physical health, as well as indirectly through lifestyle factors. A similar pattern of interaction is observed for self-rated mental health, frailty, and mortality, although there are differences in different lifestyle factors and district associations. Lifestyle factors also directly affect physical and mental components of health, frailty, and mortality. The magnitude of direct district effect is comparable to those of lifestyle and socioeconomic position.

**Conclusions/Significance:**

We conclude that district variations in health outcomes exist in the Hong Kong elderly population, and these variations result directly from district factors, and are also indirectly mediated through socioeconomic position as well as lifestyle. Provision and accessibility to health services are unlikely to play a significant role. Future studies on these district factors would be important in reducing health disparities in the older population.

## Introduction

With the ageing of populations worldwide, the major users of health services are older people. Increasing emphasis has been placed on the collection of data documenting variations or disparities in health outcomes, with a view to minimizing these disparities as part of public health improvement [1]. However collection of outcomes at the macro level has limitations, in that it is difficult to have a clear understanding of contributory factors from only ecological data. Furthermore, the fact that more resources tend to be direct at areas with poorer outcomes gives rise to cross-sectional associations linking increase spending with poor outcomes [2]. For any country or city, while it is important to document regional variations in health outcomes, in depth studies using primary data are needed to identify possible contributory factors in order to minimize disparities. Different strategies are required for different contributory factors. Provision and accessibility of health services, particularly of primary care [3], represent only one of many external factors. Others include social and psychological factors [4], [5], [6], [7], [8], the physical environment such as air pollution [9], [10], open spaces [11] and perhaps population density, noise and constant bright light constituting some of the neighbourhood factors. Individual factors include socioeconomic status [4], [8], [12], [13], [14], [15], lifestyle [16] and the life course influence in terms of early life environment [17] and life events [6], [13].

For health outcomes, other than macro indicators such as mortality, individual health descriptors such as self-rated physical and psychological health and degree of frailty are particularly pertinent to older populations. It has been pointed out that self-rated health may provide a more holistic indicator of health [8]. Documentation of the level of frailty provides an objective indicator of health in older people, encompassing physical, functional and mental health dimensions [18], [19], [20]. The use of frailty measures as a health outcome indicator has the advantage that it is a reflection of deficits in multiple domains, representing an intermediate stage between robust health and end of life. Two approaches have been used: an index derived from summation of all abnormalities [21], and an indicator based on specific characteristics such as grip strength, weight loss, exhaustion, low physical activity, low walking speed [22]. The former method has been shown to have a ceiling effect. Studies in health disparities in the older population should ideally examine a broad range of health outcomes other than mortality, and also include as many categories of contributory factors as possible. To date few studies address disparities in older populations specifically [8], [14], [23], using frailty as one of the health outcomes and examining the relative contributions of individual and environmental factors to health outcomes. Using a data set from a health survey of 4000 people aged 65 years and over living in all regions of Hong Kong, we examined regional variations in self-rated health, frailty, and 4 year mortality, and analyzed the relative contributions of lifestyle, socioeconomic status, and geographical location of residence to these outcomes. We hypothesize that lifestyle, socioeconomic status, as well as regional characteristics directly and indirectly through interactions contribute to self-rated physical and psychological health, frailty and four year mortality.

## Results

**Figure 1:**
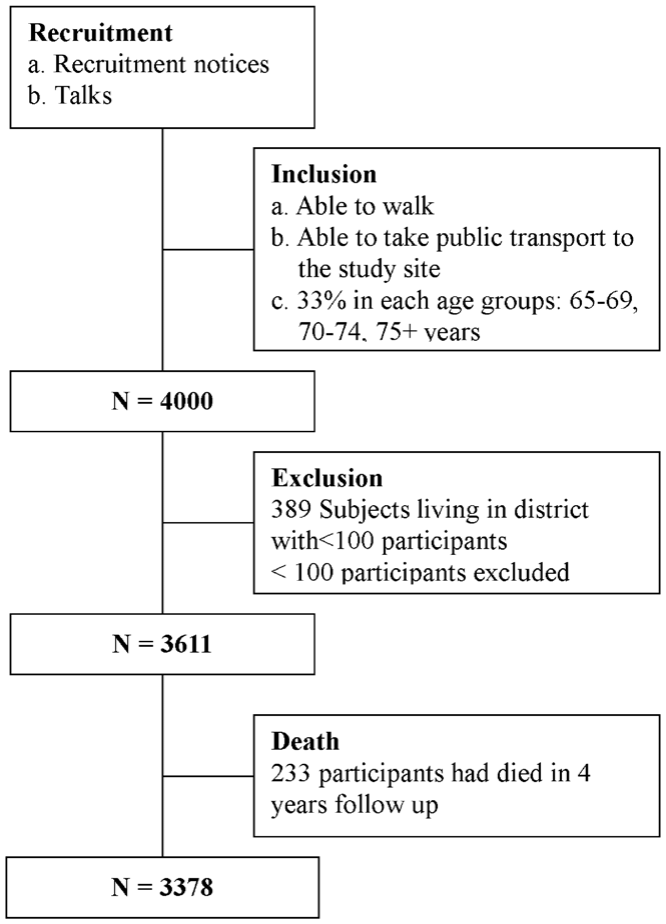
Recruitment flow chart.

The total number of subjects from 11/18 districts with > = 100 participants was 3611 (90.3% of the original sample). After four years of follow up, 233 participants had died [Fig 1]. Variations in lifestyle (DQI, PASE, smoking habit and alcohol use) with age, gender, socioeconomic status and district is shown in Table 1. Increasing age was associated with lower PASE score, less use of tobacco and alcohol. Being female was associated with better dietary quality, lower PASE score, less use of tobacco and alcohol, and higher socioeconomic position. Higher socioeconomic position was associated with lower PASE score and less use of tobacco. Using Shatin as the reference district, district variations in different components of lifestyle are observed, but do not fall into any consistent ‘healthy’ or ‘unhealthy’ pattern.

**Table 1:** Regression of DQI, PASE, smoking, alcohol use and SES in HK.

Variable	Mean (SD)/freq(%)	DQI		PASE		Smoking		Alcohol use		Higher SES in HK	
		Std. β	p-value	Std. β	p-value	Std. β	p-value	Std. β	p-value	Std. β	p-value
Age	72.52(5.21)	−0.0173	0.3146	−0.2113	**<.0001**	−0.0362	**0.0310**	−0.0882	**<.0001**	−0.0234	0.1728
Female	1847 (51.2%)	0.0846	**<.0001**	−0.1255	**<.0001**	−0.2069	**<.0001**	−0.3101	**<.0001**	0.0755	**<.0001**
Higher SES in HK	1952 (57.6%)	0.0305	0.0766	−0.0340	**0.0422**	−0.0577	**0.0006**	0.0142	0.3851	-	-
11 districts											
1. Shatin	712 (19.7%)	Ref		Ref		Ref		Ref		Ref	
2. Sai Kung	190 (5.3%)	−0.0009	0.9628	−0.0052	0.7758	−0.0248	0.1770	−0.0200	0.2610	−0.0045	0.8122
3. Tsuen Wan	156 (4.3%)	−0.0402	**0.0293**	−0.0334	0.0625	−0.0252	0.1626	−0.0065	0.7126	−0.0211	0.2522
4. Kwai Tsing	204 (5.7%)	−0.0259	0.1676	−0.0457	**0.0123**	−0.0238	0.1946	−0.0243	0.1721	−0.0087	0.6412
5. Yuen Long	179 (5.0%)	−0.0162	0.3845	−0.0611	**0.0008**	0.0000	0.9993	−0.0240	0.1756	0.0281	0.1313
6. Kowloon City	281 (7.8%)	0.0419	**0.0304**	−0.0497	**0.0083**	−0.0580	**0.0022**	−0.0085	0.6426	0.0390	**0.0437**
7. Wong Tai Sin	344 (9.5%)	−0.0319	0.1071	−0.0272	0.1575	−0.0020	0.9189	−0.0049	0.7927	0.0066	0.7379
8. Sham Shui Po	351 (9.7%)	0.0108	0.5857	−0.0366	0.0571	−0.0338	0.0814	0.0196	0.2981	0.0185	0.3490
9. Kwun Tong	600 (16.6%)	0.0107	0.6153	−0.0451	**0.0290**	−0.0363	0.0806	−0.0053	0.7945	0.0042	0.8452
10. Eastern	330 (9.1%)	0.0146	0.4625	−0.0520	**0.0070**	−0.0817	**<.0001**	0.0429	**0.0226**	0.0761	**0.0001**
11. Yau Tsim Mong	264 (7.3%)	0.0077	0.6909	−0.0566	**0.0025**	−0.0326	0.0833	−0.0145	0.4271	0.0382	**0.0467**

**Figure 2:**
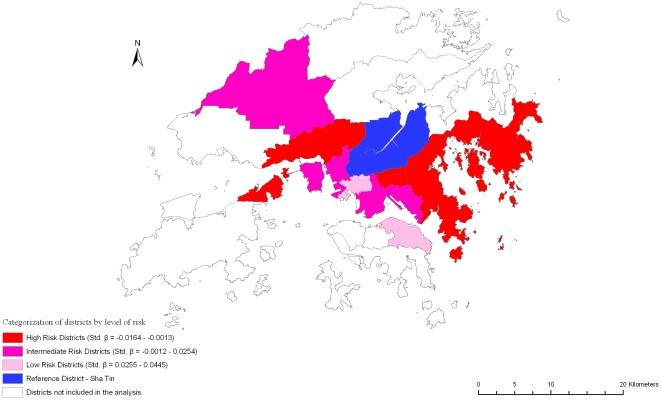
Risk variation in self-rated physical health (SF-12 physical) across districts.

**Figure 3:**
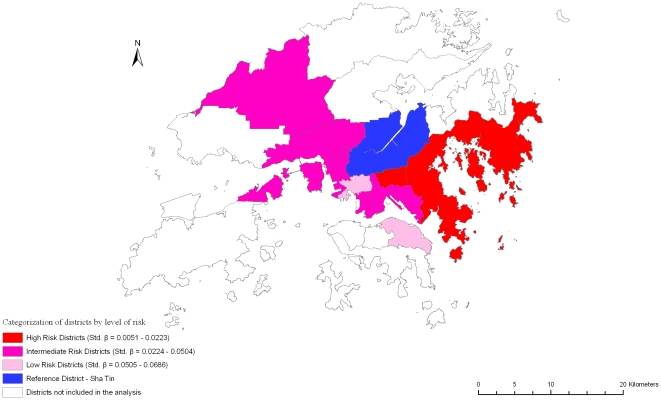
Risk variation in self-rated mental health (SF-12 mental) across districts.

**Figure 4:**
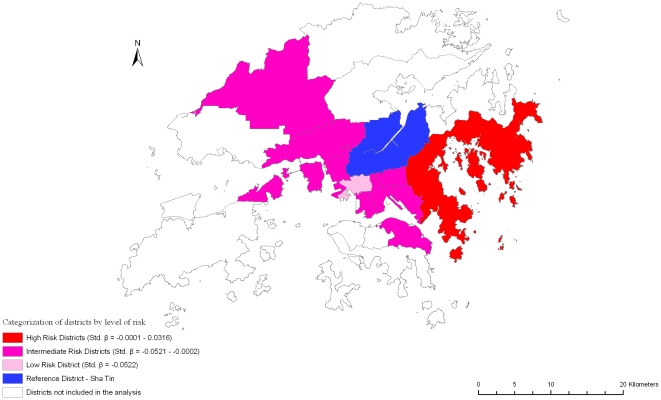
Risk variation in frailty (Log Frailty Index) across districts.

**Figure 5:**
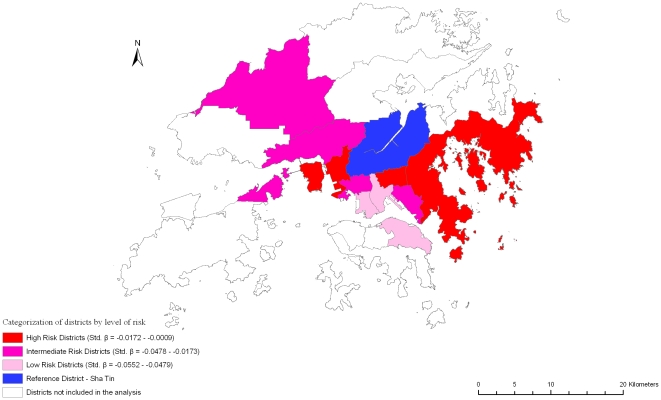
Risk variation in mortality across districts.

Variations in self-rated physical and mental health (SF-12 physical and mental scores), frailty index, and mortality are shown in Table 2. Increasing age is associated with better self-rated mental health, greater frailty, and increased mortality. Being female is associated with poorer self-rated physical and mental health, greater frailty, but decreased mortality. Better dietary quality (higher DQI) is associated with better self-rated physical and mental health, less frailty and decreased mortality. Similar results are observed with higher physical activity level, with the exception of a non-significant association with self-rated mental health. Smoking is associated with less frailty, while alcohol use was associated with better self rated physical and mental health, and less frailty. Higher socioeconomic position is associated with better outcomes overall. District variations in all four outcomes were observed, but again the variations do not fall into any consistent pattern representing areas with poor or good outcomes among all the health indicators measured (Fig 2, Fig 3, Fig 4, Fig 5).

**Table 2:** Regression of SF12-physical, SF12-mental, Log (Frailty index) and death.

Variable	Mean (SD)/freq(%)	SF12-physical		SF12-mental		Log(Frailty index)		Death	
		Std. β	p-value	Std. β	p-value	Std. β	p-value	Std. β	p-value
Age	72.52(5.21)	−0.0272	0.1091	0.0526	**0.0027**	0.1899	**<.0001**	0.1319	**<.0001**
Female	1847 (51.2%)	−0.2103	**<.0001**	−0.0566	**0.0023**	0.0737	**<.0001**	−0.1620	**<.0001**
DQI	64.39(9.57)	0.0689	**<.0001**	0.0693	**<.0001**	−0.0862	**<.0001**	−0.0537	**0.0016**
PASE	91.13(42.20)	0.0953	**<.0001**	0.0222	0.2105	−0.1070	**<.0001**	−0.0512	**0.0034**
Smoking	249 (6.9%)	0.0278	0.1052	−0.0335	0.0581	−0.0715	**<.0001**	0.0111	0.5242
Alcohol use	458 (12.7%)	0.0409	**0.0201**	0.0378	**0.0373**	−0.0838	**<.0001**	−0.0132	0.4607
Higher SES in HK	1952 (57.6%)	0.0993	**<.0001**	0.0701	**<.0001**	−0.0633	**0.0001**	−0.0360	**0.0332**
11 districts									
1. Shatin	712 (19.7%)	Ref		Ref		Ref		Ref	
2. Sai Kung	190 (5.3%)	−0.0076	0.6728	0.0051	0.7833	0.0316	0.0780	−0.0009	0.9622
3. Tsuen Wan	156 (4.3%)	−0.0013	0.9421	0.0504	**0.0061**	−0.0083	0.6371	−0.0271	0.1339
4. Kwai Tsing	204 (5.7%)	0.0213	0.2387	0.0393	**0.0355**	−0.0163	0.3652	−0.0062	0.7379
5. Yuen Long	179 (5.0%)	0.0238	0.1855	0.0370	**0.0466**	−0.0211	0.2383	−0.0263	0.1497
6. Kowloon City	281 (7.8%)	0.0197	0.2918	0.0378	0.0507	−0.0065	0.7271	−0.0552	**0.0037**
7. Wong Tai Sin	344 (9.5%)	−0.0164	0.3901	0.0223	0.2581	−0.0002	0.9928	−0.0072	0.7113
8. Sham Shui Po	351 (9.7%)	0.0422	**0.0272**	0.0686	**0.0005**	−0.0522	**0.0059**	−0.0271	0.1617
9. Kwun Tong	600 (16.6%)	0.0254	0.2149	0.0298	0.1595	−0.0238	0.2422	−0.0173	0.4066
10. Eastern	330 (9.1%)	0.0445	**0.0204**	0.0617	**0.0019**	−0.0169	0.3748	−0.0479	**0.0141**
11. Yau Tsim Mong	264 (7.3%)	0.0096	0.6069	0.0434	**0.0235**	−0.0117	0.5237	−0.0523	**0.0056**

**Figure 6:**
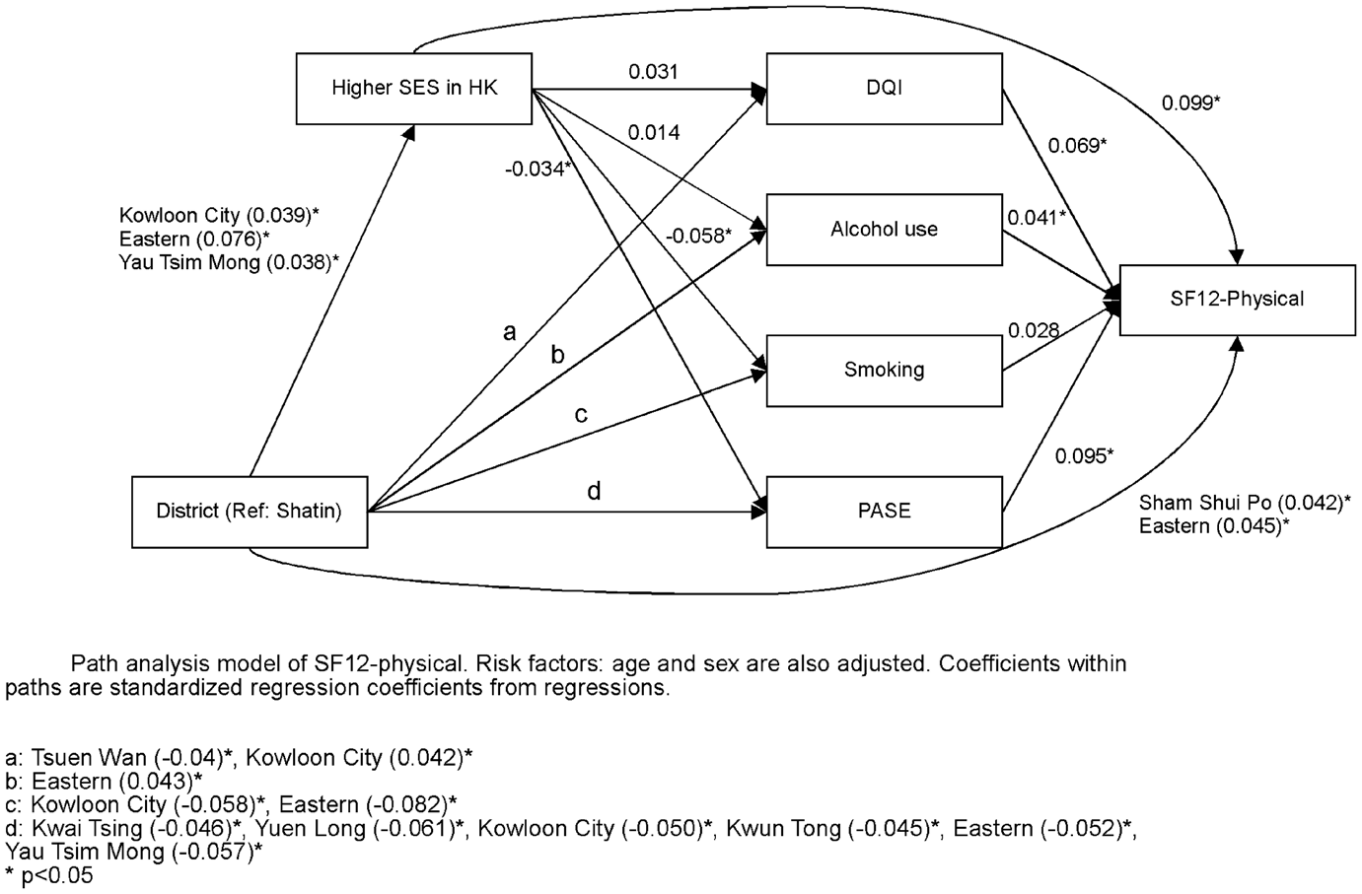
Path analysis model of SF12-physical.

**Figure 7:**
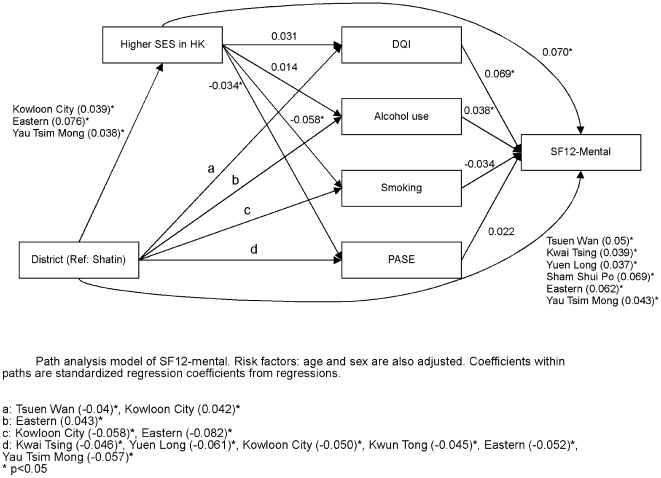
Path analysis model of SF12-mental.

**Figure 8:**
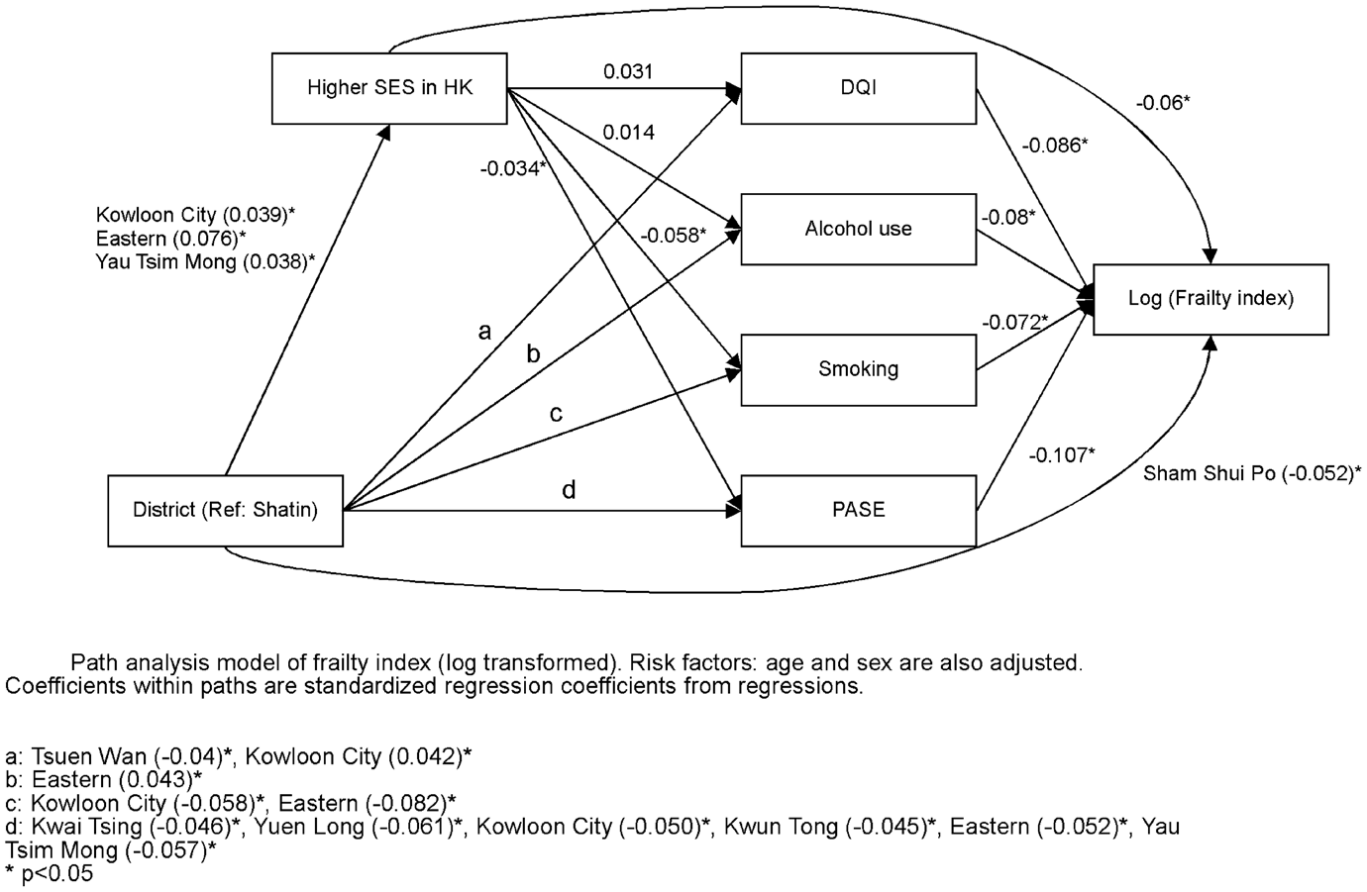
Path analysis model of frailty index (log transformed).

**Figure 9:**
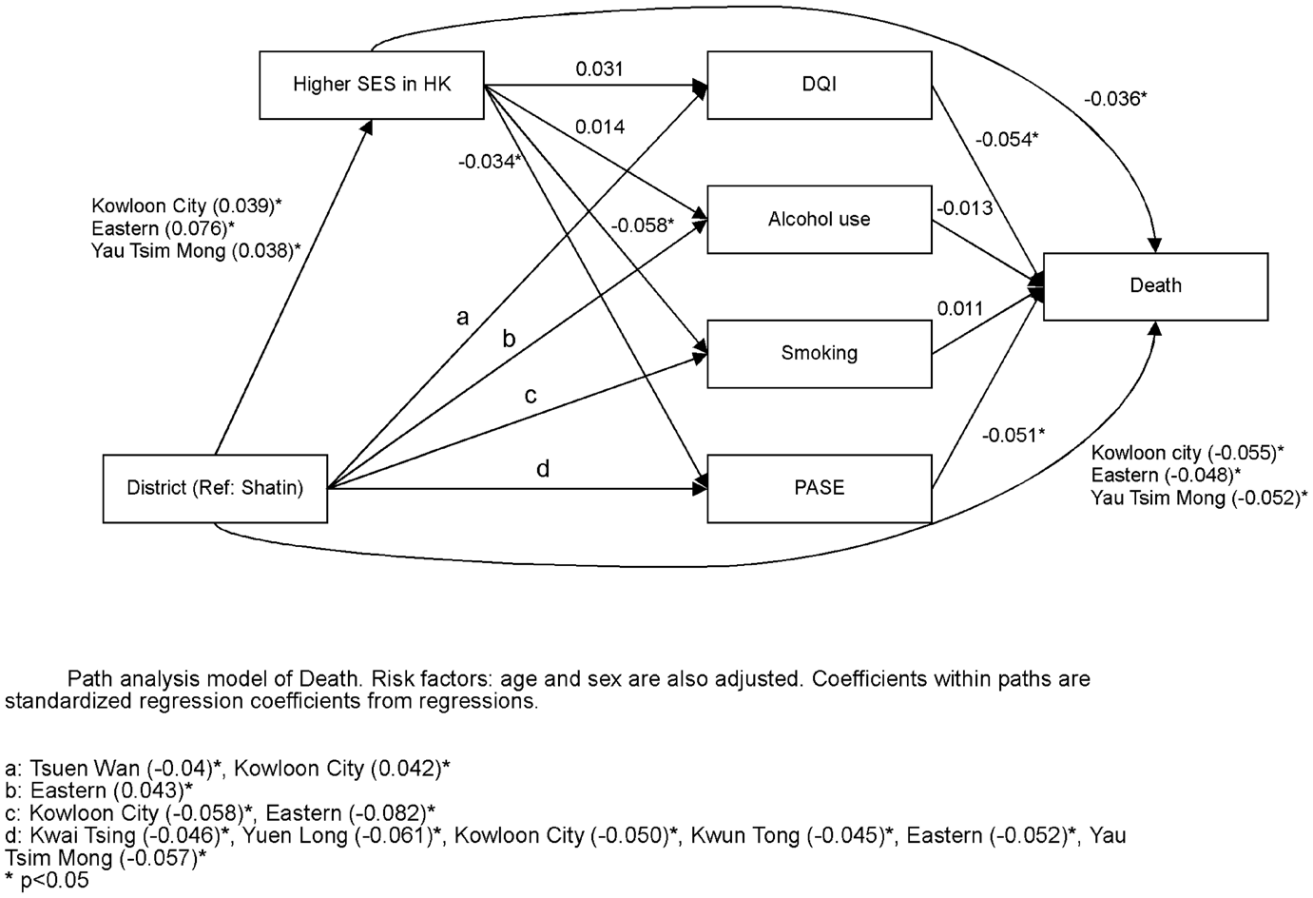
Path analysis model of Death.

Path analysis was carried out in order to determine the direct or indirect effect of district, socioeconomic and lifestyle factors on each of the four health outcomes. Fig 6 shows that district variations directly affect self-rated physical health, and also exert an effect through socioeconomic position as well as lifestyle factors. Socioeconomic position in turn directly affects self-rated physical health as well as indirectly through lifestyle factors. A similar pattern of interaction is observed for self-rated mental health (Fig 7), frailty (Fig 8), and mortality (Fig 9), although there are differences in different lifestyle factors and district associations. The magnitude of direct district effect is comparable to those of lifestyle and socioeconomic position.

We also attempted to capture the impact of other geographical parameters such as air pollution index, population density, and net land area listed as open spaces (un-inhabited areas) using government provided data. However these were not available for all districts examined here, and little association with health outcomes was noted for districts with available data.

## Discussion

Analysis of this dataset shows that even within a city of 1050 km^2^ with a population of 6.7 million people, small area variations in health outcomes exist among the elderly aged 65 years and over. District of residence, socioeconomic position and lifestyle factors directly as well as indirectly affect self-rated physical and mental health, frailty and four year mortality in a complex manner.

Previous studies on health disparities placed great emphasis on the type and accessibility to health services [24]. Comparisons of health outcomes in populations with different health care systems and accessibility would need to take into account individual lifestyle and socioeconomic position. When other factors are taken into account, the relationship between health outcomes and health care accessibility may not be very strong. A study of those aged <65 years in the UK showed only a weak correlation between geographical access to services and rates of morbidity in rural areas [25]. An analysis of health disadvantage of rural populations in Australia showed that while rural location plays a major role in determining the nature and level of access to and provision of health services, it does not always translate into health disadvantage [7]. A comparison of health outcomes among US, Europe and England with distinctly different healthcare systems showed that healthcare systems, wealth, as well as lifestyle contribute to variations [14]. In this study we did not examine service accessibility as a contributory factor, since there is a comprehensive range of health and social services accessible to all irrespective of income in Hong Kong. The very efficient public transport system ensures easy accessibility. While there are minor district variations in the provision of health services in terms of number of doctors and hospital beds, there is no restriction of access between districts, such that patients may choose to attend whichever health facilities outside their district of residence for shorter waiting times. Furthermore, although primary care is largely provided by private practitioners, the Accident and Emergency Departments of district hospitals effectively also function as primary care facilities for those who cannot afford to pay private consultation fees.

The relationship between lifestyle and socioeconomic position, and the four health outcomes has been observed in other studies. A ‘healthy’ diet and physical activity have been shown to reduce mortality [16]. This study also shows that a healthy lifestyle is also associated with reduced frailty and better self rated physical and mental health. The association between use of tobacco and alcohol consumption and better health outcomes may be explained by avoidance of smoking and alcohol among those in poorer health, since cross-sectional data are used. Variations in health outcomes by socioeconomic determinants have also been documented in numerous previous studies, although variations may be less pronounced in older compared with younger or middle age populations [5], [8], [26], [27].

In this study we used self-rated socioeconomic position, as this measure captures psychological factors related to objective socioeconomic measures, which may be more powerful in examining social determinants of health compared with objective indicators alone [15]. Subjective status has been considered a more precise measure of social position and an example of the relationship between health and social hierarchy [28] and to have better correlation with psychological and physical functioning compared with objective social indicators [29]. Self rated socioeconomic status could be a strong predictor of self rated health, with associations similar to traditional socioeconomic status measures [30].

The inclusion of frailty as a health outcome measure has not been reported previously in the context of disparities, since frailty is a characteristic of older people and there have been relatively few studies examining the elderly population in particular. The results show that a healthy lifestyle is also associated with reduced frailty, similar to the relationship for mortality [16], independent of socioeconomic position. The finding that lower socioeconomic position is associated with increased frailty is consistent with previous reports of increased ill-health and disability with lower socioeconomic status [27].

Although district of residence may indirectly contribute to variations in health outcomes through socioeconomic position and lifestyle, the district factor alone directly contribute to the variation. These may consist of neighbourhood characteristics such as social support, accessibility to transport, leisure and medical facilities, and safety, these being composite of indicators of neighbourhood deprivation [31]. Other factors include environmental pollution (air, noise and excessive light), crowdedness or lack of space. These factors likely exert effect on health outcomes partly through psychological mechanisms medicated via the neuroendocrine system to increase allostatic load [32]. For example in a subset of this population, we noted that those residing in densely populated districts compared with those in districts with many open spaces have shorter white blood cell telomere length, a reflection of greater cumulative cellular oxidative stress [33]. Air pollution has also been linked to hospital admissions for respiratory diseases [34], and may be contributory to variations in health outcomes. Future studies to delineate the components of this district effect are needed to inform efforts to reduce small area variations in health outcomes. Other efforts to reduce such disparities could include targeted promotion of healthy lifestyles according to district. Socioeconomic disparities would need to be address in the population as a whole. For example the current cohort of elderly have lower educational attainment, but future cohorts will have higher educational levels as a result of compulsory and free schooling for at least nine years.

There are limitations in this study. Large database studies have the inherent limitations in that the study was not designed to address a single specific question, but is essentially hypothesis generating. Therefore conclusions would need to be regarded in this context. Except for mortality, other health outcomes are examined with associated factors in a cross sectional design, so that causal relationships may not be drawn. The participants may not be representative of their district of residence, since they responded to the initial invitation to the study centre and may be regarded as volunteers. Bias may occur in either direction: those who were health conscious and those who had health problems may both have been more likely to respond. The cohort as a whole was of a higher educational standard compared with the general Hong Kong population. No district based population norms were available for comparison. Furthermore, the number of participants from each of the 18 districts varied widely, with some districts having fewer than twenty participants. We arbitrarily examined only those districts with > = 100 participants, so that only 11/18 districts were included. We did not taken into account the life course dimension, inspite of literature documenting the impact of early life environment on subsequent health outcomes later in life [35], and also the impact of exposure to adverse life events [6], [8]. We did not examine district factors in depth. The strength of this study lies in the broad range of health outcomes measured including frailty, an important indicator in older populations, the detailed lifestyle information, the use of self rated socioeconomic position to take into account the psychological aspects, and the use of primary data which allows the linkage of contributory factors to health outcomes at the individual level.

Inspite of the limitations, we can conclude that district variations in health outcomes exist in the elderly population, and these variations result directly from district factors (yet to be determined) and also indirectly mediated through socioeconomic position as well as lifestyle. Provision and accessibility to health services are unlikely to play a significant role. Future studies on these district factors would be important in reducing health disparities in the older population.

## Materials and Methods

### The Setting

Hong Kong is a Special Administrative Region of China situated in the South Coast of the Guangdong province, with a population of 6.7 million in an area of 1,050 km^2^. Approximately 12% of the population are aged 65 years and over [36]. Geographically it is separated into 18 administrative districts. Each of these districts is represented by a District Board and a district management committee which has been established in Hong Kong since 1982 under the District Administration Scheme (http://www.had.gov.hk). For the purpose of effective coordination and responsiveness of government policies on district needs and problems, district offices communicate and advise the government on issues concerning the local well-being such as the provision of services and facilities, the promotion of community, recreational and cultural activities, and environmental improvements within the districts. Apart from this administration purpose, the boundaries of the 18 districts have also been constructed to delineate relatively homogeneous spatial units which are distinguished from one another both in terms of demography profile, socio-economic indicators as well as the provision of healthcare services across these districts (http://www.ha.org.hk/qeh/statistics_report.pdf; http://www.bycensus2006.gov.hk/File Manager/EN/Content_962/06bc_dcd.pdf). Health service delivery in Hong Kong is divided into public and private sectors. Hospital services at secondary and tertiary levels are largely provided by the public sector and subsidized by the local government. Hospital Authority (HA) and Department of Health (DOH) provide medical treatment and rehabilitation services to patients through public hospitals, general outpatient and specialist clinics and outreach services throughout the territory, targeted at serving socially disadvantaged and vulnerable groups. The private sector plays a complementary role by providing only 6 percent of total hospital care. With respect to curative primary care services, private practitioners of Western medicine account for more than half of the market share. However, people believe that public hospitals provide quality healthcare services to citizens at reasonable prices. Due to this inadvertently flawed incentive system, there is an over-reliance on hospitals such that people also visit the accident and emergency departments (AED), general and specialist outpatient clinics (GOPC and SOPC) in the public sector for primary care services.

### Participants

Four thousand men and women aged 65 years and over living in the community in all regions of HK were invited to attend a health check carried out in the School of Public Health of the Chinese University of Hong Kong, by placing recruitment notices in community centers for the elderly and housing estates. Several talks were also given at these centers explaining the purpose, procedures and investigations to be conducted. The inclusion criteria were that all participants should be able to walk or take public transport to the study site at the University teaching hospital in Shatin. Participants were volunteers, and the aim was to recruit a sample such that approximately 33% were in each of these age groups: 65–69, 70–74, 75+ years. Compared with the general population in this age group, participants had higher educational level (12–18% v. 3–9% with tertiary education in the age groups 80+, 75–79,70–74, and 65–69 years) [36]. The study was approved by the Clinical Research Ethics Committee of the Chinese University of Hong Kong, which required informed consent to be obtained. All participants signed the study consent forms.

### Procedures

A questionnaire containing information regarding demographics, socioeconomic status in terms of educational level and maximum life-time income, medical history, smoking, alcohol intake, physical activity level, and dietary intake was administered by an interviewer. Physician diagnosed disease was obtained by self-report. Self-rated socioeconomic status was assessed by asking participants to place a mark on a picture of an upright ladder with ten rungs, with the lowest rung being the most undesirable and the highest the most desirable state with respect to their standing in the community (community ladder). This is a subjective measure of social status developed by the John D and Catherine T MacArthur Research Network on Socioeconomic Status and Health, and has been associated with key health outcomes in various population surveys of different cultural and ethnic groups [29]. Participants were also asked to rate themselves by placing a mark on a picture of another ladder, the top rung representing people who have the most money, the most education, and the most respected jobs, and the bottom rung representing people at the other extreme (Hong Kong ladder). Data on the community ladder and the Hong Kong ladder were available for 3834 men and 3759 women, respectively.

Physical activity level was assessed using the Physical Activity Scale of the Elderly (PASE) [37]. This is a 12-item scale measuring the average number of hours per day spent in leisure, household and occupational physical activities over the previous 7 day-period, and had been used previously in other epidemiological studies in Hong Kong. The test-retest reliability over a 3–7 week interval was 0.75 (95%CI 0.69–0.80). Internal consistency was adequately high, with an intra-class correlation coefficient ranging from 0.65–0.91 [38], [39] It has been shown to correlate well with health status and physiologic measures, energy expenditure, mid-thigh muscle area per bodyweight [37], [38], as well as ambulatory physical activity measured by Actigraph monitors in the elderly [39]. No studies reported on the responsiveness to change, although changes in this measure had been noted with time during longitudinal follow-up of the subjects in the cohort. Activity weights for each item were determined based on the amount of energy expended, and each item score was calculated by multiplying the activity weight by activity daily frequency. A summary score of all the items reflected the daily physical activity level. A higher score represents greater physical activity level. Health-related quality of life was assessed using the SF-12 validated in a Chinese population [40].

Dietary intake was assessed at baseline using a food frequency questionnaire (FFQ), and mean nutrient quantitation per day was calculated using food tables derived from McCance and Widdowson [41] and the Chinese Medical Sciences Institute [42]. The FFQ had been validated with the basal metabolic rate calculation and the 24-hour sodium/creatinime and potassium/creatinine analysis [43]. The FFQ consisted of seven categories; Bread/pasta/rice; vegetables; fruits; meat/fish/eggs; beverages; dimsum/snacks; soups; and oil/salt/sauces. Each subject was asked to complete the questionnaire – the food item, the size of each portion, the number of times of consumption each day and each week. Portion size was explained to subjects using a catalogue of pictures of individual food portions. The amount of cooking oil was estimated according to the method of preparing different foods: 0.2 tablespoon for steaming fish or stir frying half a portion of meat. For food items consumed less than once per week, information was obtained for consumption pattern over one year and the quantitation per day or week adjusted accordingly.

The Diet Quality Index-International (DQI-I) was used to assess the quality of diet [44], since it is an indicator of dietary patterns in relation to health. The DQI-I has also been used to evaluate the quality of diet in a Chinese population [45]. Four major aspects of the diet are assessed: variety, adequacy, moderation and overall balance, each with subcomponents. The range is 0–100, with high score indicating high quality. In this study, we did not have sufficient information to calculate the category of empty-calorie foods under the aspect ‘moderation’. Therefore, the range of score for moderation was 0–24 instead of 0–30, and the DQI-I total score was 0–94 instead of 0–100. The reproducibility correlation for the 2 FFQ scores was 0.72. Correlations between scores for each of the 2 FFQs and diet records were 0.66 (FFQ-1) and 0.72 (FFQ-2). DQI-R scores from FFQ-2 were directly correlated with plasma biochemical measurements of α-carotene (*r* = 0.43, *P* = 0.0005), β-carotene (*r* = 0.35, *P* = 0.005), lutein (*r* = 0.31, *P* = 0.005), and α-tocopherol (*r* = 0.25, *P* = 0.05) and were inversely correlated with plasma total cholesterol (*r* = −0.22, *P* = 0.05) [46].

The Chinese version of DQI has also been developed and validated in a Chinese community (N = 7450, recruited from the 1991 China Health and Nutrition Survey) using cross-sectional 3 day diet record and anthropometric data [47]. The total DQI score was significantly correlated with food and nutrient intakes, BMI, urban residence and income. In addition, the DQI pattern scores correlated with DQI components and weight status.

Frailty was quantified using the frailty index (FI). The majority of the ageing population spend a variable period before death in a state of declining function, described as the frailty syndrome, representing an excess of deficits over assets in a dynamic state of balance, covering physical, functional, psychological, nutritional and social domains [48], [49], [50]. The concept of a summation measure of deficits as a measure of frailty, the frailty index (FI), had been developed in a Canadian population [18], [51] and found to be applicable to the Chinese population [21]. The validity of the FI was supported by its association with mortality [18], and health outcomes such as hospitalization, functional and cognitive decline [20], and was also influenced by social determinants [19]. Following the principle of calculating of FI in previous studies [21], [51], abnormality in each parameter was assigned a score of 1, and summated. The FI was calculated as the total score divided by the maximum total score 47 (Appendix S1). Since the distribution of the FI was skewed, log transformed FI was used in subsequent analysis.

The following items from the health check questionnaire were used to construct a list of deficits, used for calculating the frailty index: self-reported health, history of falls in the past 12 months, history of osteoporotic fractures, presence of back pain limiting activities, clumsiness in walking, clumsiness using hands, the number of prescription medications and any difficulties with performing activities of daily living (walking two to three blocks outside on level ground, climbing up 10 steps without resting, preparing own meals, doing heavy housework such as scrubbing floors or washing windows, and doing own shopping for groceries or clothes). The presence or absence of disease was based on subjects' report of diagnosis by their doctors. Depressive symptoms were assessed using the Geriatric Depression Scale [52] with a score ≥8 representing depressive symptoms, validated in elderly Chinese subjects [53]. Cognitive impairment was assessed using the Cognitive Screening Instrument for Dementia (CSID) with a cut off point of ≤28.4 [54].

The following measurements were carried out: height, weight, time taken to walk 6 meters, grip strength, blood pressure, and ankle brachial index (ABI). Body weight was measured with subjects wearing a light gown, by the Physician Balance Beam Scale (Healthometer, Illinois, USA). Height was measured by the Holtain Harpenden standiometer (Holtain Ltd, Crosswell, UK). Body mass index was calculated by dividing the weight in kg by the square of the height in meters. Grip strength was measured using a dynamometer JAMAR hand dynamometer (5030JI, Sammons Preston, Bolingbrook, IL). The average reading for two readings on right and left side was used. Blood pressure was measured in the supine portion using a mercury sphygmomanometer and the averages of two readings were taken. Duplicate measures of supine blood pressure in right arm and both ankles were performed using a standard mercury sphygmomanometer and an 8-MHz Doppler probe (Pocket Doppler Model 841-A, Parks Medical Electronics, Inc. ALOHA, OR, USA). The ABI was calculated for each leg by dividing the posterior tibial systolic pressure in each lower extremity by the upper extremity pressure. The current standard for diagnosing peripheral vascular disease is defined as an ABI of less than 0.90. An ABI of less than 0.90 is 95% sensitive and 99% specific for angiographically diagnosed peripheral arterial disease [55]. The lowest ankle-arm index of the two was used to determine the extent of ischemic disease.

Mortality after 4 years was ascertained from the Government Death Registry.

### Statistical Analysis

Since there were variable numbers of participants from the 18 districts, we arbitrarily excluded districts with less than 100 participants (seven districts) as the number may be too small for detailed analysis. Shatin was used as the reference district as the study centre was located in Shatin. The relationship between contributory factors and each of the health outcomes is examined using path analysis. This is an extension of simple regression modeling of dependent variable on independent variables to show associations between variables. DQI, PASE, smoking and alcohol use were regressed on age, sex, socioeconomic status and districts. Four outcome variables: physical health (SF12-physical), mental health (SF12-mental), log-transformed frailty index and death were then regressed on all other independent variables in separate models. Standardized regression coefficients were presented within paths. All statistical analyses were performed using the statistical package SAS, version 9.1 (SAS Institute, Inc., Cary, North Carolina). An α level of 5% was used as the level of significance.

## Supporting Information

Appendix S1 Frailty index.
(0.04 MB DOC)
