# Supplementary material for: Relative Contributions of Geographic, Socioeconomic, and Lifestyle Factors to Quality of Life, Frailty, and Mortality in Elderly
Source: PLoS One. 2010 Jan 19;5(1):e8775. doi: 10.1371/journal.pone.0008775 (PMC2808254; doi:10.1371/journal.pone.0008775)
Supplement: Appendix S1 — Frailty index. (0.04 MB DOC) [file pone.0008775.s001.doc]

Appendix S1

**Frailty index**

| Items | Score |
| --- | --- |
| CSID abnormal | Yes=1 |
| GDS>=8 | Yes=1 |
| Self rated health (Poor or very poor) | Yes=1 |
| No. of disease* | Max=18 |
| Past 12m fall>1 | Yes=1 |
| No. of impairments of IADL | Max=5 |
| No. of fracture | Max=4 |
| Clumsy walking | Yes=1 |
| Clumsy using hands | Yes=1 |
| Back pain: limited activities | Yes=1 |
| No. of medication use | Max=7 |
| BMI<18.5kg/m^2^ | Yes=1 |
| ABI<0.9 | Yes=1 |
| Grip strength <10 percentile (M<23, F<15) | Yes=1 |
| 6 meter walk time >10 percentile (M>7.65, F>8.63) | Yes=1 |
| SBP>140 | Yes=1 |
| DBP>90 | Yes=1 |
| **Total** score | **Max=47** |

*Diseases include: diabetes, thyroid disease, osteoporosis, stroke, Parkinson’s disease, hypertension, MI, angina, congestive heart failure, COPD, Prostatitis, glaucoma, cataracts, gastrectomy, arthritis, kidney stone, cancer, back pain

**Frailty index = score of frailty items/ 47**
